# Pandemics and the English Language: Concepts Critical for Conversing About COVID-19

**DOI:** 10.20411/pai.v7i2.542

**Published:** 2022-11-10

**Authors:** Neil S. Greenspan, Guillermo A. Pereda

**Affiliations:** 1 Department of Pathology, Case Western Reserve University, Cleveland, Ohio

**Keywords:** SARS-CoV-2, coronavirus, COVID-19, immunity, vaccine, mRNA, immune escape, immune evasion, cause of death, pathogen, relational variable

## Abstract

We consider the multiple senses of several key terms that are used to discuss the ongoing COVID-19 pandemic and clarify meanings of the corresponding concepts. Topics addressed include: 1) the meaning of immunity to an infectious agent in varying medical and scientific contexts, 2) the scientific factors that influenced the rapid generation and clinical implementation of safe and effective vaccines for COVID-19, 3) the difference between mutational abrogation of reactivity with B- or T-cell antigen receptors (immune escape) versus active interference with host immune mechanisms mediated by gene products encoded within the genome of the infectious agent (immune evasion), 4) the different ways by which the COVID-19 pandemic has “caused” deaths, and 5) briefly, the challenge of precisely defining the term *pathogen*.

## INTRODUCTION

Although it is more than 75 years since George Orwell published his widely known and often admired essay, “Politics and the English Language” (PEL), his indictment of then-current writing for the broader public remains highly relevant [1]. In PEL, he severely criticized hackneyed phrases and imagery, the lack of precision in the use of words, and pretentious language. Of equal importance, Orwell forcefully supported the argument that however arrived at, widespread degradation in the use of language to convey nuance and complexity in ideas can then favor further decline in the quality of thought in an unfortunate cycle that harms politics, social interactions, and culture.

The shortcomings in communication Orwell highlighted are still with us in many contexts. Our focus is on the ways that language can be unclear or misleading in connection with coverage of the COVID-19 pandemic, a topic of broad interest for now more than 2½ years. Since non-scientists, as well as biomedical researchers and physicians with varying areas and extents of expertise are routinely commenting about viruses and their capabilities, antibodies, vaccines, epitopes, and various forms of immunity, the potential complexities in the meanings of these and other terms are not always fully recognized or adequately conveyed.

We are interested in the underlying scientific concepts and mechanisms and the importance of precision in the language employed to discuss these ideas and phenomena. Therefore, we think it is a suitable moment to discuss what some of these key words or phrases are intended (by a writer or speaker) or interpreted (by a reader or listener) to mean. In succession, we discuss a series of terms and concepts pertinent to the pandemic and that are routinely commented on in newspapers and magazines as well as on radio and television and in podcasts. We acknowledge that the topics we cover are illustrative and do not constitute a comprehensive exploration of the words and concepts that are relevant for understanding and conversing about COVID-19.

Before discussing the language specific for the COVID-19 pandemic, we offer some generalizations based on the first author's roughly 45 years of immersion in immunology and related fields of biomedical science. Summarizing this experience, a tentative conclusion is that in too many instances, individuals communicating about biomedical matters use words and concepts without having thought sufficiently about the precise meanings they attach to them [2, 3]. This supposition may bear some resemblance to the ideas that motivated Socrates to probe the thought processes of his fellow citizens in ancient Athens. Specifically, we suggest that individuals often fail to clarify for themselves precisely where to draw the defining boundaries for the categories these terms are intended to reference.

## THE MEANINGS OF *IMMUNITY*

In discussions of COVID-19 and SARS-CoV-2, physicians, journalists, and scientists often comment on the presence, absence, or extent of immunity to the virus. However, in different contexts, *immunity* can take on different senses such that the distinctions among these varying meanings may be clinically or scientifically significant.

Below, are several plausible different meanings for *immunity* in the context of a pandemic:

1. There is an immune response of some sort that exhibits specificity for a pathogen (ie, an infectious agent that causes tissue damage and/or perturbs physiologic function) or for pathogen-associated antigens.
2. There is an immune response of some sort that exhibits specificity for a pathogen or for pathogen-associated antigens expected to be targets of protective responses.
3. There is an immune response that can specifically target and eliminate the pathogen but where it is unclear if the magnitude of this response is sufficient to confer protection from infection.
4. There is an immune response that can specifically target and eliminate the pathogen but where it is unclear if the magnitude of this response is sufficient to confer protection from severe disease, hospitalization, or death.
5. There is *actual* protection from infection.
6. There is *actual* protection from symptomatic disease but not infection.
7. There is *actual* protection from severe disease but not infection or less severe disease.
8. There is *actual* protection from death but not infection or disease of any lesser degree of severity.

*Immunity*, although less commonly, could also mean protection against transmission, as has been considered in vaccine development for malaria caused by *Plasmodium falciparum* (Pf), generally the cause of the most severe forms of disease caused by parasites of any of the *Plasmodium* species that infect humans. In the case of vaccination against Pf, immunity to gametocytes, the sexual stage of the parasite, protects not the direct vaccine recipient but rather the individuals to whom the vaccine recipient might transmit gametocytes via mosquito vectors. Although we do not currently have any such vaccines approved and in clinical use, such a vaccine remains plausible.

As implied above, protective immunity can encompass more than the mere production of antigen-specific antibodies or T cells. Laboratory methods may indicate whether an individual has produced either of these mediators of immunity in response to a pathogen but fail to definitively indicate whether these elements of immunity are of sufficient magnitude to be effective at preventing clinical manifestations of infection through destruction and/or clearance of the pathogen. The mere presence of an immune response, even of the right sort and specificity, does not necessarily equate to any particular degree of pathogen destruction or clearance or any particular level of protection against different unwelcome outcomes. Of course, it is well-known that host immune and inflammatory responses elicited by infectious agents can be of such intensity that they cause damage to host tissues, as is the case for SARS-CoV-2 [4].

Generally speaking, T cells alone cannot provide a complete barrier to infection or detectable clinical effects, whereas antibodies can sometimes provide a degree of immunity able to prevent any obvious clinical manifestations even if infection is not absolutely prevented. While T cells may be limited in their ability to prevent disease, they have been credited with effectively clearing a pathogen from host cells and tissues to resolve an infection, for example, for infections caused by influenza A viruses [5–8]. Frequently, humoral and cell-mediated immune mechanisms, along with innate immune mechanisms, will contribute to pathogen clearance and recovery from infection.

Differences in the intended meanings of *immunity* may stem from professionals' differing roles and goals. For example, physicians, public health workers, and public servants may feel the need to simplify their language when communicating with the general public. This sort of action is taken with the intention of informing people who to varying degrees may lack knowledge about infectious diseases and agents, the dangers associated with infection, and relevant safety protocols. On the other hand, news reporters and journalists may feel compelled to use language and concepts that while more accessible compromise accuracy to some extent. These actions can blur the nuances in meaning of important terms such as *immunity*. **It is imperative that scientists and biomedical researchers remain cognizant of these complexities of communication and be explicit about how a given term is being used in their written or spoken statements.**

## THE SOURCE(S) OF OUR GOOD FORTUNE IN THE RAPID PRODUCTION OF SAFE AND EFFECTIVE COVID-19 VACCINES

How is it that effective and safe vaccines for COVID-19 were produced in about 1 year from the beginning of the outbreak caused by SARS-CoV-2? For most pathogens, vaccine developers have historically required several years or more from the time an infectious agent is identified as the cause of a disease to produce a safe and effective vaccine formulation [9, 10]. Therefore, some have attributed the extraordinarily rapid development of vaccines for COVID-19 to the unique talents of those involved, referring to them as heroes [11, 12]. These contributors include the research scientists and corporate specialists in a range of roles that are necessary to transform laboratory discoveries into clinical-scale vaccines for which the Food and Drug Administration has granted either emergency use authorization or approval [13]. There is certainly some truth to this assessment.

But we should note that researchers and company scientists of comparable skill and dedication have been working on other vaccines for much longer without comparable or even any success. For example, in the case of HIV-1, scientists have been working to develop a vaccine for almost 40 years with no clearly effective formulation identified so far [14, 15]. Similarly, the same caliber of scientists and other personnel have been working to develop vaccines for Pf for at least 60 years [16]. The first vaccine approved for malaria just gained approval within the past year, and it is unlikely to be more effective than the best vaccines being used for COVID-19 in the United States and other countries [17].

If we have a future epidemic with a totally new virus, or other infectious agent, for which the antigens mutate much more rapidly than do those of SARS-CoV-2 or for which we are uncertain about which antigens can reliably elicit protective immunity, development of a safe and effective vaccine could take far longer. Even with people who were as skilled and as hard working as those who generated the COVID-19 vaccines, we might wait years for an effective vaccine to such a pathogen despite existing mRNA vaccine technology, established adenovirus vectors, and other methods pressed into service for vaccine production in the current pandemic. Although SARSCoV-2 may be a “resourceful” and “devious” pathogen in several ways, it presents a relatively simple challenge in terms of basic vaccine design.

So why was success so rapid for the vaccine targeting SARS-CoV-2? We wish to focus in some detail on the scientific factors that contributed to the rapid pace of development for COVID-19 vaccines and refer the interested reader to Kuter *et al* [18] for discussion of factors relating primarily to modifications of the testing and production processes involved in the vaccine pipeline.

**Figure 1.**
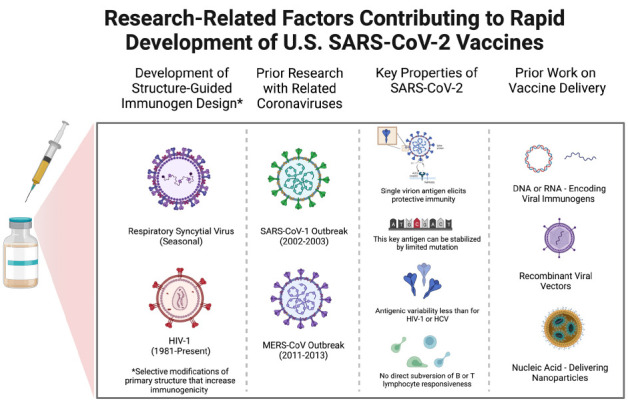
**Scientific factors contributing to the relatively rapid development of safe and effective SARS-CoV-2 vaccines.** The schematic diagrams from left to right allude to the roles of prior research on structure-guided vaccine design, previous work focused on the immunologic attributes of coronaviruses similar in key respects to SARS-CoV-2, critical features of SARS-CoV-2 itself, and past efforts to develop the use of nucleic acids that encode immunogens to be produced by recipient cells as well as viral vectors and nanoparticles for vaccine delivery to host cells.

We focus on 3 primarily scientific reasons, 2 of which are related, for the ability to so quickly identify a successful immunization approach (see Figure 1). First, the outbreak of severe acute respiratory syndrome (SARS) in 2002-2003 caused by a coronavirus we could now term SARS-CoV-1, which, as the name suggests, is closely related to SARS-CoV-2, prompted a great deal of high-quality research [19]. The current work on SARS-CoV-2 built on these studies, similar work on the Middle East Respiratory Syndrome (MERS) virus, and on studies of less virulent coronaviruses associated with the common cold. This research has been proceeding in a number of labs on the less virulent coronaviruses for decades [20].

SARS-CoV-1 is closely related to SARS-CoV-2 in many attributes relevant for understanding immunity and designing vaccines. For instance, studies from over 15 years ago showed that antibodies against the SARS-CoV-1 spike protein could protect experimental animals against infection and disease [21–23]. Investigators learned how to stabilize the SARS-CoV-1 spike protein, and later the MERS virus spike protein, through mutation to enhance immunogenicity and elicit protective antibodies able to neutralize the virus [24, 25].

In this work, Barney Graham and Jason McLellan and their colleagues at the National Institute of Allergy and Infectious Diseases and later McLellan's group at the University of Texas determined that a small change in the amino acid sequence of the spike protein could stabilize the conformation of the molecule that exists prior to binding of the spike protein to the receptor, ACE2, on the host cell membrane [25].

By immunizing against the spike protein in its stabilized conformation, it is more likely that the elicited antibodies will prevent the virus from successfully entering host cells, ie, neutralize the virus. This key research performed with the SARS-CoV-1 and other coronavirus spike proteins was readily applied to the SARS-CoV-2 spike protein.

Second, SARS-CoV-2, like SARS-CoV-1, presents an unusually straightforward challenge for vaccine development compared to infectious agents such as HIV-1, hepatitis C virus (HCV), Pf, or *Mycobacterium tuberculosis* (Mtb). These pathogens are associated with much greater genome sequence diversity (HIV-1 and HCV), possess the ability to become latent in host cells (HIV-1 and Mtb), infect and inhibit the function of key cells involved in immune responses (HIV-1 and Mtb), have multiple life stages with different sets of antigens at each stage (Pf), or produce a multitude of antigens (Mtb) such that the best targets for protective immunity are difficult to determine with certainty [15, 26, 27].

For the coronaviruses, a single antigen encoded by a single viral gene suffices as a target for protective immunity elicited by vaccination and mediated by antibodies, which is the form of immunity that has been studied most extensively after vaccination [28]. The prior experience with SARS-CoV-1 and MERS virus, in conjunction with extraordinary similarities between these 2 coronaviruses and SARS-CoV-2, provided a strong rationale for immediately focusing vaccine development efforts on the SARS-CoV-2 spike protein [29, 30].

The third reason for the rapid success involves the 2 to 3 decades of both academic and corporate research that paved the way for successful immunization with nucleic acids or viral vectors containing genes encoding 1 or more relevant pathogen-associated proteins. Immunization by administering “naked” nucleic acids encoding immunogenic proteins capable of eliciting immunity was initiated first by Liu and colleagues with DNA and later extended to RNA by Kariko and Weissman [31, 32].

Also relevant is the work begun years ago on the use of nanoparticles of various compositions for delivery of nucleic acids for one or another purpose [33, 34]. In fact, as noted by Robert Langer, a co-founder of Moderna, in a recent interview, as of early 2020 when the threat of COVID-19 was first recognized in the United States, Moderna, BioNtech, and Curevac already had initiated clinical trials with a variety of mRNA-based vaccines for other pathogens [35]. Work with a variety of viral vectors for vaccination, including adenoviruses like those used for 2 of the widely used COVID-19 vaccines, goes back 25 years [36, 37].

There is now growing advocacy for developing schemes able to facilitate extremely rapid (ie, within 100 days) production of vaccines against newly identified pathogens with pandemic potential [38]. Others have advocated for using the genomic sequence of a newly identified viral pathogen with pandemic potential to directly produce a vaccine mRNA encoding the presumed key immunogen based on analogies to known pathogens in the same virus family [39]. We believe these ideas, although intended to address real and important needs, should be viewed with a measure of caution due to still incompletely fathomed complexities of both vaccine safety and effectiveness.

## VIRUS *ESCAPE* VERSUS VIRUS *EVASION* OF IMMUNITY

During a surge of cases caused by the Delta variant of SARS-CoV-2, some commenting on the increasing case numbers suggested that this version of virus was better able to evade the immune response. Others have referred to the same phenomenon as immune escape by the Delta variant.

**Figure 2.**
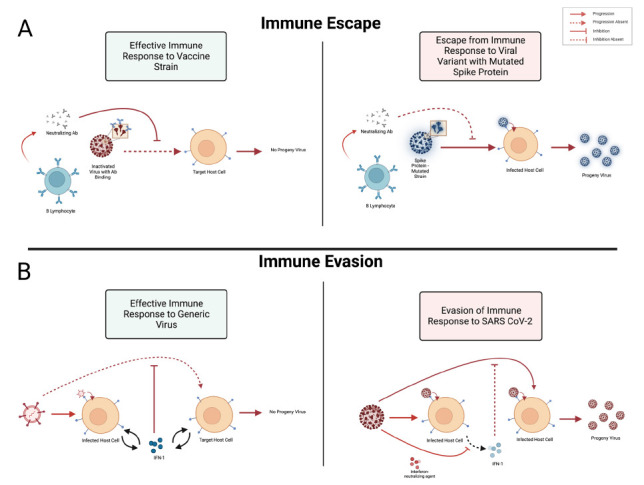
**Schematic illustration of the key differences between the mechanisms of what we suggest corresponds to *immune escape* (top) versus what corresponds to *immune evasion* (bottom).** Our view is that *immune escape* should refer to one or more mutations in the genome of a virus or other infectious agent that meaningfully decrease recognition of a key pathogen-associated antigen by antibodies or T-cell receptors. In contrast, *immune evasion* should correspond to the situation where 1 or more gene products encoded in the genome of the infectious agent function to inhibit or otherwise undermine relevant immunological processes of the host, thereby hindering clearance or destruction of that agent by those host mechanisms. The example of viral proteins that interfere with the production or functioning of type I interferons is used to illustrate the concept of immune evasion.

We propose to make a distinction between 1) the processes by which viruses, or other pathogens, evolve on relatively short time scales so as to reduce the effectiveness of antigen-specific immune mechanisms dependent on either B or T lymphocytes, and 2) the processes by which these infectious agents produce gene products that directly inhibit or otherwise interfere with the functions of host gene products that mediate adaptive or innate immune pathways (Figure 2).

These latter virus-initiated mechanisms will typically be the result of evolution and selection that took place over longer time intervals.

Our preference is to reserve *escape* for those instances in which, for example, a mutation in SARSCoV-2 spike protein renders a previously effective neutralizing antibody completely ineffective or at least much less able to inhibit the ability of the virus to infect host cells. Escape is a process requiring as little as a single point mutation typically occurring, as noted above, on relatively short evolutionary time scales.

On the other hand, we would suggest that we label the mechanisms by which SARS-CoV-2-encoded proteins disrupt normal pathways through which host proteins cooperate to destroy or eliminate the virus as examples of *evasion*. For example, it has been reported that SARS-CoV-2 produces several non-structural proteins that reduce the production by host cells of molecules known as type 1 interferons. These latter host-derived molecules alter host cells in ways that make those cells more resistant to virus infection [40–42]. This host pathway is generally classified as a type of innate immunity, meaning in part that it is not antigen-specific.

Which precise words are used is ultimately less important than the ideas embodied by the distinction between *escape* and *evasion* for which we advocate. For example, a term that could be considered as an alternative for *evasion* of host immunity is *subversion*, which like *evasion* connotes an active process.

Irrespective of the words selected, the distinction can matter because different types of interventions may better address one sort of process versus the other. Irrespective of the words chosen to refer to these 2 processes, it is important to note that both escape and evasion, and other mechanisms manifested by some infectious agents, can be employed more or less simultaneously by at least some viral or other pathogens.

## DEATHS “CAUSED” BY COVID-19

Every day, the *New York Times* lists statistics pertaining to the COVID-19 pandemic [43]. Among these numbers is a daily death toll attributed to infection by SARS-CoV-2. This prompts a question: What counts as a death due to infection by SARS-CoV-2 versus any other causal factor? Please note that throughout the following discussion we acknowledge the enormous significance of every death for family and friends of the deceased regardless of what factor or factors can be viewed as causal and that the motivation for making the arguments presented below is to permit more accurate assessments of risks associated with this virus. An ability to compare relative risks posed by different infectious agents is a potential first step to limiting deaths due to infectious disease.

Elsewhere, in a different context, we have come to grips with the necessity, also noted by others before us, that to enumerate any entity it is necessary to have a definition of that entity that is clear enough to decide, literally, what counts and what does not [3]. This realization is accompanied by the likelihood that there may be different contexts that are best served by somewhat different definitions. Therefore, there may be reason to provide multiple answers to what sounds like a simple question. Nevertheless, many people, perhaps a majority of people, will expect there to be a single definitive answer. We must remember that reality feels no obligation to make life simple.

So, it is not unreasonable to, along with journalists, list any death of an individual infected by SARS-CoV-2 as a death caused by COVID-19. But what if a person infected by SARS-CoV-2 has a pre-existing and severe lung condition and ultimately dies? Did the virus, the pre-existing condition, or both cause the death? Should this sort of case be included with deaths of individuals with no known pre-existing conditions? At what magnitude of impairment in pulmonary function, or function of any critical organ (eg, heart, kidneys, or liver), if at all, does the balance of causal responsibility tip from the virus to the pre-existing organ pathology? If someone gets infected and dies soon thereafter, before the infection can substantially degrade lung or other organ function, how should such a death be classified? These and other questions that could be asked suggest the potential complexities of what sound initially like simple matters of enumeration.

How should we classify deaths of people with an ongoing serious medical condition for which they could not get timely treatment due to the changes in the availability of medical care caused by a surge in cases of COVID-19? These lives are lost because of a different condition but likely earlier than would otherwise have been the case specifically due to effects of the pandemic, an indirect but highly significant influence. This speculation is supported by the increased excess mortality in the United States during 2020 [44–46].

It is important to explain precisely what criteria are being employed when a given individual death is included or not in a given number because otherwise it will become difficult to track whether a given pathogen, such as SAR-CoV-2, is becoming more or less lethal. Lack of clarity regarding which instances of death count and which do not as pathogen-caused will also make reliable comparisons among viral or other pathogens difficult or impossible.

## WHAT IS A PATHOGEN?

Finally, everyone agrees that infection by SARS-CoV-2 can cause tissue damage, reduced tissue or organ function, and in some cases death. That is generally what infectious disease experts, microbiologists of every subfield, immunologists, and other biomedical researchers and physicians mean when they refer to this coronavirus as a pathogen.

Of course, as is well-known, some people infected by SARS-CoV-2 exhibit no or minimal symptoms and have no longer-term sequelae. Others may be asymptomatic in the acute phase but nevertheless have significant symptoms weeks and months later. Of course, many infected individuals have obvious symptoms during the acute phase of the infection including those who are sufficiently severely affected to be admitted to the hospital, be admitted to the hospital intensive care unit, be intubated, or die from what appear to be impairments caused directly by the virus or by inflammatory or immunological responses elicited by the virus.

So, it is no surprise that like other pathogens, even pandemic pathogens only exhibit virulence in some hosts and to varying degrees. This reality raises the question: “What are we then to label a microbe that exhibits virulence much less frequently but not never?” For example, in a study in mice from the Belkaid lab [47], infection by a recognized pathogen, *Yersinia pseudotuberculosis*, seems to make it possible for a microbe normally found in the human gut and regarded as a source of benefit, lactobacillus, to cause a chronic infection in mesenteric lymph nodes. Commenting on the Belkaid article in *Science* [48], Carl Nathan suggests that perhaps the prior infection with *Y. pseudotuberculosis* may provide a context within which lactobacilli become virulent in some sense, ie, pathogenic.

We cannot in this space comprehensively address such a large and complex topic as the nature of pathogens and pathogenicity. Our limited purpose here is merely to hint at the scale of complexity that is associated with such a familiar and widely used concept [49]. We also wish to highlight the inherent lack of clarity frequently associated with such critical terms, which we view as a notion of broader applicability within and beyond biomedical science [50]. An additional critical point to which we call attention about the label *pathogen* is that it refers *not* to an attribute intrinsic to a microbe or parasite, but to an attribute of the relationship between a particular microbe or parasite and a particular host. Thus, pathogenicity is a relational variable, like antibody affinity, which applies to a particular pairing of antibody and antigen under defined conditions and is not an inherent attribute of an antibody [51, 52].

## CONCLUSION

Pandemics are characterized by great complexity in multiple respects. Discussions of pandemics typically employ many terms and numbers the meanings of which are inadequately explained or explored. Greater clarity of expression is to be strongly desired to enhance understanding of pandemic infectious diseases both among pertinent specialists and the general public, to facilitate further investigation, and to avoid costly errors in policy or medical practice.
